# Two New Alkaloids from *Fusarium tricinctum* SYPF 7082, an Endophyte from the Root of *Panax notoginseng*

**DOI:** 10.1007/s13659-018-0171-0

**Published:** 2018-06-18

**Authors:** Wen-Jie Sun, Hong-Tao Zhu, Tian-Yuan Zhang, Meng-Yue Zhang, Dong Wang, Chong-Ren Yang, Yi-Xuan Zhang, Ying-Jun Zhang

**Affiliations:** 1 State Key Laboratory of Phytochemistry and Plant Resources in West China, Kunming Institute of Botany, Chinese Academy of Sciences, Kunming, 650201 People’s Republic of China; 2 University of Chinese Academy of Sciences, Beijing, 100049 People’s Republic of China; 3 Shenyang Pharmaceutical University, Shenyang, 110016 People’s Republic of China; 4 Yunnan Key Laboratory of Natural Medicinal Chemistry, Kunming Institute of Botany, Chinese Academy of Sciences, Kunming, People’s Republic of China

**Keywords:** *Fusarium tricinctum* SYPF 7082, Endophytic fungus, Alkaloids, *Panax notoginseng*, Inhibition on NO production

## Abstract

**Abstract:**

*Panax notoginseng* (Araliaceae) is a famous traditional Chinese medicine mainly cultivated in Yunnan and Guangxi provinces of China. Two new alkaloids, rigidiusculamide E (**1**) and [-(*α*-oxyisohexanoyl-*N*-methyl-leucyl)_2_-] (**2**), together with two known ones, (−)-oxysporidinone (**3**) and (−)-4,6′-anhydrooxysporidinone (**4**) were isolated from the mycelia culture of *Fusarium tricinctum* SYPF 7082, an endophytic fungus obtained from the healthy root of *P. notoginseng*. Their structures were determined on the basis of extensive spectroscopic analyses. Compounds **1**–**4** were tested for their inhibitory effects against NO production on Murine macrophage cell line, and the new compound **2** showed significant inhibitory activity on NO production with the IC_50_ value of 18.10 ± 0.16 μM.

**Graphical Abstract:**

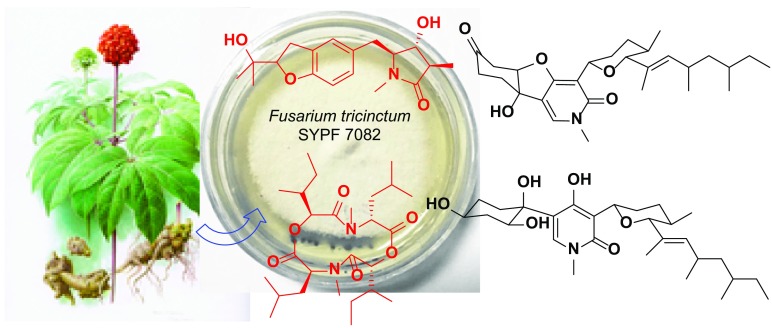

**Electronic supplementary material:**

The online version of this article (10.1007/s13659-018-0171-0) contains supplementary material, which is available to authorized users.

## Introduction

*Panax notoginseng* (Burk.) F. H. Chen (Araliaceae), known as Sanqi or Tianqi in China, is a famous traditional Chinese medicine [1], with a broad spectrum of pharmacological effects, e.g., anti-atherosclerotic [2], hemostatic and wound healing [3], antioxidant [4], anti-inflammatory [5], hypoglycemic and anti-hyperlipidemia [6], neuroprotective [7], and anti-tumor [8] activities. The plant has been cultivated and domesticated for approximately 400 years, mainly in Yunnan and Guangxi provinces, China. Continuous cultivation of *P. notoginseng* in the same field will led it to be attacked vulnerably by various soil-borne pathogens, like fungi, bacteria and nematodes [9]. The rhizospheric and endophytic fungal communities are considered not only of vital importance for plant health and soil fertility, but also to have positive effects on plant resistance to diseases and insects. These factors might be useful for the biological control of continuous cropping of *P. notoginseng* [10].

*Fusarium* species, a group of filamentous fungi with a number of plant pathogens in it [11], are widely distributed in soil, plants and plant-products. The secondary metabolites of which could be great resources for finding new compounds with a variety of biological activities [12]. For example, previous studies on *F. tricinctum* led to the identification of neosolaniol monoacetate and visoltricin from the strains of field-loss peanuts [13] and wheat kernels [14], and tricinonoic acid and tricindiol, enniatins and fusarielins, and fusartricin from the endophytic fungi from *Rumex hymenosepalus* [15], *Aristolochia paucinervis* [16, 17], and *Salicornia bigelovii* [18], respectively.

During the research on the formation mechanism of continuous cropping obstacles of *P. notoginseng*, two new alkaloids, rigidiusculamide E (**1**) and [-(*α*-oxyisohexanoyl-*N*-methyl-leucyl)_2_-] (**2**), together with two known ones (**3** and **4**) were identified from the mycelia culture of *F. tricinctum* SYPF 7082, an endophytic fungus isolated from the healthy root of *P. notoginseng*. Their structures were determined by extensive spectroscopic analyses. Moreover, the inhibitory activities of compounds **1**–**4** against NO production in Murine macrophage cell line were evaluated. This paper describes the isolation, structure elucidation and results of bioassay.

## Results and discussion

**Fig. 1:**
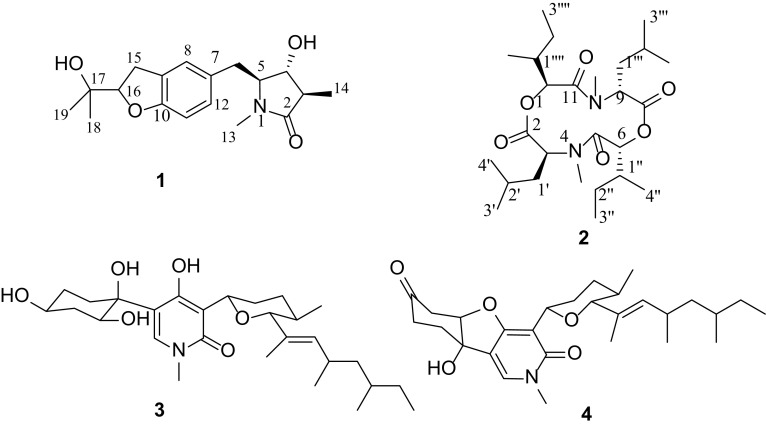
Structures of compounds **1**–**4** from *F. tricinctum* SYPF 7082

The EtOAc extract of the mycelia culture of *F. tricinctum* SYPF 7082, isolated from the root of *P. notoginseng* was applied to repeated column chromatography (CC) over MCI-gel CHP20P and silica gel, followed with semi-preparative HPLC, to afford four alkaloids (**1**–**4**) (Fig. 1). Two of them, **1** and **2** were new compounds.

Rigidiusculamide E (**1**), a colorless oil, had a molecular formula of C_18_H_25_NO_4_ on the basis of HRESIMS (*m/z* 342.1673 [M+Na]^+^, calcd. 342.1676) and NMR data (Table S1), requiring seven degrees of unsaturation. The IR spectrum showed the presence of hydroxyl group (3419 cm^−1^), amide (1669 cm^−1^) and benzene ring (1492 and 1442 cm^−1^). The ^13^C NMR and DEPT data of **1** exhibited 18 carbon resonances assignable to four methyls (*δ*_C_ 8.6, 24.4, 26.6, 27.9), two methylenes (*δ*_C_ 30.9, 32.5), four methines (*δ*_C_ 42.7, 64.5, 69.1, 90.0), one oxygenated quaternary carbon (*δ*_C_ 72.0), one carboxylic carbon (*δ*_C_ 176.2), and six aromatic carbons [*δ*_C_ 109.2 (CH), 126.0 (CH), 128.9 (CH), 128.0 (C), 129.2 (C), 158.6 (C)] arising from a tri-substituted benzene ring. The ^1^H NMR spectrum displayed the existence of three singlet [*δ*_H_ 1.15, 1.28, 2.80 (each s)] and one doublet (*δ*_H_ 1.13, d, *J* = 7.2 Hz) methyls, and a set of aromatic protons [*δ*_H_ 6.66 (1H, d, *J* = 8.4 Hz), 6.98 (1H, d, *J* = 8.4 Hz), and 7.06 (1H, s)] from an ABX coupled system (Table 1). These NMR features are closely related to those of rigidiusculamide D, an alkaloid reported previously from *Albonectria rigidiuscula* [19]. However, instead of the oxygenated quaternary C-3 (*δ*_C_ 75.1, qC) in rigidiusculamide D, an aliphatic methine (*δ*_C_ 42.7, CH) was present in **1**, suggesting that compound **1** was an analog of rigidiusculamide D without oxygen-substitution at C-3 position.

**Table 1:** ^1^H (600 MHz) and ^13^C (150 MHz) NMR spectroscopic data for compounds **1**–**2** (in CDCl_3_, *δ* in ppm and *J* in Hz)

No.	**1**		No.	**2**	
	*δ*_C_	*δ*_H_		*δ*_C_	*δ*_H_
2	176.2, C		2	171.1, C	
3	42.7, CH	2.35, m	3	83.8, CH	5.01, d (9.6)
4	69.1, CH	3.94, dd (9.0, 4.8)	4	30.8, N-CH_3_	3.02, s
5	64.5, CH	3.52, m	5	172.2, C	
6	32.5, CH_2_	2.95, dd (13.2, 4.2) 2.84, dd (13.2, 4.2)	6	58.0, CH	4.84, dd (15.0, 7.2)
7	129.2, C		8	171.1, C	
8	126.0, CH	7.06, s	9	83.8, CH	5.01, d (9.6)
9	128.0, C		10	30.8, N-CH_3_	3.02, s
10	158.6, C		11	172.2, C	
11	109.2, CH	6.66, d (8.4)	12	58.0, CH	4.84, dd (15.0, 7.2)
12	128.9, CH	6.98, d (8.4)	1′	40.9, CH_2_	1.94, m 1.59, m
13	27.9, *N*-CH_3_	2.80, s	2′	26.4, CH	1.58, m
14	8.6, CH_3_	1.13, d (7.2)	3′	22.6, CH_3_	1.05, d (6.0)
15	30.9, CH_2_	3.10, dd (15.7, 9.0) 3.24, dd (15.7, 9.0)	4′	23.6, CH_3_	1.05, d (6.0)
16	90.0, CH	4.53, t (9.6)	1′′	38.3, CH	1.90, m
17	72.0, C		2′′	26.2, CH_2_	1.25, m 1.48, m
18	26.6, CH_3_	1.28, s	3′′	11.5, CH_3_	0.96, t (7.5)
19	24.4, CH_3_	1.15, s	4′′	16.7, CH_3_	1.08, d (6.0)
			1′′′	40.9, CH_2_	1.94, m 1.59, m
			2′′′	26.4, CH	1.58, m
			3′′′	22.6, CH_3_	1.05,d (6.0)
			4′′′	23.6, CH_3_	1.05,d (6.0)
			1′′′′	38.3, CH	1.90, m
			2′′′′	26.2, CH_2_	1.25, m 1.48, m
			3′′′′	11.5, CH_3_	0.96, t (7.5)
			4′′′′	16.7, CH_3_	1.08, d (6.0)

**Fig. 2:**
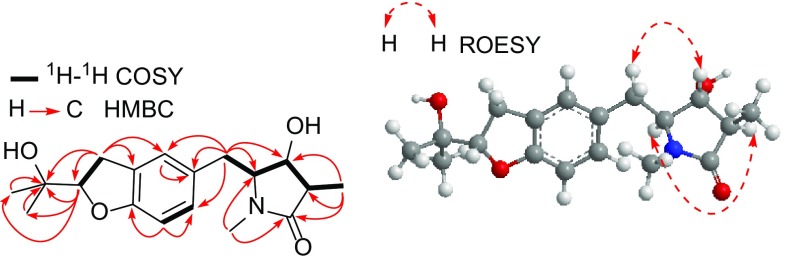
Key ^1^H-^1^H COSY, HMBC and ROESY correlations of **1**

The structure of **1** was further confirmed by 2D NMR experiments. In the ^1^H-^1^H COSY spectrum, three partial structures of -C_(14)_H_3_-C_(3)_H-C_(4)_H(O)-C_(5)_H(N)-C_(6)_H_2_-, -C_(11)_H=C_(12)_H- and -C_(15)_H_2_-C_(16)_HO- were observed. The HMBC correlations from H_2_-15 (*δ*_H_ 3.10) to C-8 (*δ*_C_ 126.0), C-9 (*δ*_C_ 128.0), and C-10 (*δ*_C_ 158.6), and from H-16 (*δ*_H_ 4.53) to C-10 indicated the presence of dihydrobenzofuran ring. Moreover, HMBC correlations from the *N*-methyl protons at δ_H_ 2.80 to C-2 (*δ*_C_ 176.2) and C-5 (*δ*_C_ 64.5), from H_3_-14 (*δ*_H_ 1.13), H-3 (*δ*_H_ 2.35) and H-4 (*δ*_H_ 3.94) to C-2 revealed the existence of 3-methylpyrrolidin-2-one moiety. Other HMBC correlations (Fig. 2) from H_2_-6 (*δ*_H_ 2.84) to C-4 (*δ*_C_ 69.1), C-5 (*δ*_C_ 64.5), C-7 (*δ*_C_ 129.2), C-8 (*δ*_C_ 126.0), and C-12 (*δ*_C_ 128.9) confirmed the planar structure of **1** as shown in Fig. 1, with 4-hydroxy-1,3-dimethylpyrrolidin-2-one ring and a dihydrobenzofuran in molecule.

In the ROESY spectrum of **1**, correlations of H-3 with H-5 (*δ*_H_ 3.52, m), and of H-4 with H-6a (*δ*_H_ 2.95, dd, *J* = 13.2, 4.2 Hz) and H-6b indicated that H_3_-14 and H_2_-6 were at the same side, while H-3 and H-5 were on the opposite orientation of the 4-hydroxy-3-methylpyrrolidin-2-one ring (Fig. 2), thereby established the relative configurations of **1**. On the basis of the above evidence, the structure of **1** was deduced as shown.

**Fig. 3:**
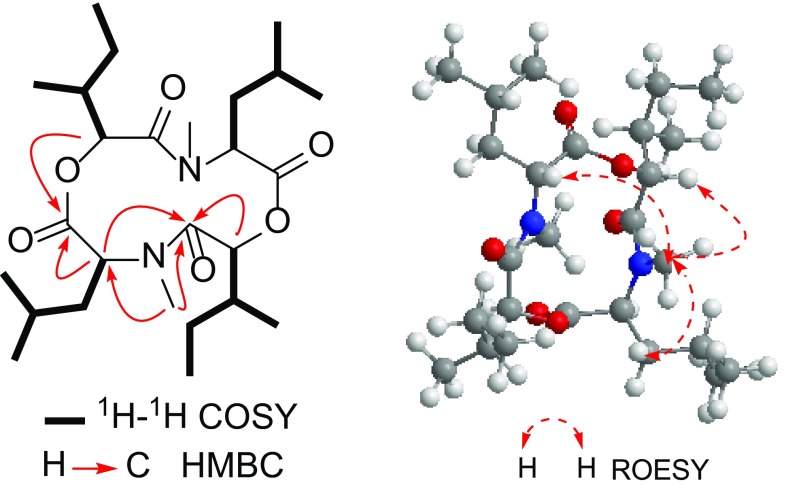
Key ^1^H-^1^H COSY, HMBC and ROESY correlations of **2**

[-(*α*-Oxyisohexanoyl-*N*-methyl-leucyl)_2_-] (**2**), obtained as colorless crystal, had a molecular formula of C_26_H_46_N_2_O_6_, deduced from the HRESIMS (*m/z* 505.3247 [M + Na]^+^, calcd. 505.3248), with five degrees of unsaturation. The IR spectrum showed the presence of carboxyl ester (1758 cm^−1^) and amide (1657 cm^−1^) groups. The ^13^C NMR and DEPT spectra of **2** exhibited 13 carbon resonances, arising from five methyls (*δ*_C_ 11.5, 16.7, 22.6, 23.6, 30.8), two methylenes (*δ*_C_ 26.2, 40.9), four methines (*δ*_C_ 26.4, 38.3, 58.0, 83.8), and two carboxylic carbons (*δ*_C_ 171.1, 172.2). The ^1^H NMR spectrum displayed the existence of one singlet (*δ*_H_ 3.02, s), two doublet [*δ*_H_ 1.05, 1.08 (each d, *J* = 6.0 Hz)] and one triplet (*δ*_H_ 0.96, t, *J* = 7.5 Hz) methyls, and two oxymethines [*δ*_H_ 4.84 (dd, *J* = 15.0, 7.2 Hz); 5.01 (d, *J* = 9.6 Hz)] (Table 1). The above-mentioned data accounted for all the ^1^H and ^13^C NMR resonances and the molecular formula suggested that **2** had a symmetrical structure. The ^1^H-^1^H COSY spectrum showed the existence of two partial structures, -CHO-CH-(CH_3_)-CH_2_-CH_3_ and -CHN-CH_2_-CH-(CH_3_)_2_ (Fig. 3). In the HMBC spectrum of **2**, correlations from *N*-methyl proton (*δ*_H_ 3.02) to C-3 (*δ*_C_ 83.8) and C-5 (*δ*_C_ 172.2), from H-3 (*δ*_H_ 5.01) to C-2, C-5 (*δ*_C_ 172.2), C-1′ (*δ*_C_ 40.9) and C-2′ (*δ*_C_ 26.4), from H-6 (*δ*_H_ 4.84) to C-5, C-8 (*δ*_C_ 171.1), *N*-methyl (*δ*_C_ 30.8), C-1′′ (*δ*_C_ 38.3) and C-2′′ (*δ*_C_ 26.2) (Fig. 3), established the fragment structures of *N*-methyl-leucyl and α-oxyisohexanoyl moieties, and the gross structure of **2** when considering of its symmetrical structure. The ROESY correlations of *N*_4_-CH_3_ with H-1′, H-6 and H-9 indicated that these protons on the same face of the cyclodipeptide ring, thereby established the relative configurations of **2** (Fig. 3). Therefore, the structure of **2** was determined as shown.

The known compounds **3** and **4** were identified to be (−)-oxysporidinone (**3**) [20] and (−)-4,6′-anhydrooxysporidinone (**4**) [21] by comparing their spectroscopic data with literature values. Both of them were isolated for the first time from *F. tricinctum*.

The inhibitory activities of compounds **1**–**4** against NO production on Murine macrophage cell line were evaluated by Griess assay [22]. Compound **2** showed inhibition of NO production with the IC_50_ value of 18.10 ± 0.16 μM, while compounds **1**, **3** and **4** were inactive at the concentration of 25 μM.

## Experimental Section

### General Experimental Procedures

Optical rotations were measured on a HORIBA SEPA-300 high-sensitive polarimeter, and UV spectra were recorded on a Shimadzu UV2401A ultraviolet–visible spectrophotometer. Infrared spectroscopy (IR) spectra were obtained on a Bio-Rad FTS-135 series spectrometer. HRESIMS date were obtained using API QSTAR Pular-1 spectrometer. ^1^H and ^13^C NMR spectra were acquired with Bruker DRX-600 spectrometer, using CDCl_3_ as solvent and TMS as an internal standard. Chemical shifts were reported in units of *δ* (ppm) and coupling constants (*J*) were expressed in Hz. Column chromatography (CC) were carried out over silica gel (200–300 mesh, Qingdao Haiyang Chemical Co., Ltd., Qingdao, China) and MCI-gel CHP20P (75–100 μm, Mitsubishi Chemical Co. Ltd., Tokyo, Japan). An Agilent series 1260 (Agilent Technologies) were used for semi-preparative HPLC with an Agilent ZORBAX SB-C18 column (5 μm, 250 × 9.4 mm), with flowing rate of 3 mL/min.

### Fungal material

The fungal strain used in this work was isolated from the healthy root of *P. notoginseng*, which was collected from Wen-Shan district, Yunnan province of China (104^o^19′17.2′′/23^o^31′48.9′′). The RNA sequence data derived from this strain has been submitted and deposited in GenBank with the accession number MG930027. BLAST search results revealed that the isolate belongs to the genus *Fusarium* and had a close relationship (99% identity) with *Fusarium tricinctum* (KR071697). A voucher specimen (SYPF 7082) has been deposited at the School of Life Science and Biopharmaceutics, Shenyang Pharmaceutical University.

### Fermentation and Isolation

The strain of *Fusarium* sp. SYPF7082 was cultivated on potato dextrose agar (PDA) at 25 °C for seven days. Fermentation was carried out in 300 Erlenmeyer flasks (250 mL) each containing 90 g rice. Sterile water (100 mL) was added to each flask, and the contents were autoclaved at 121 °C for 30 min. After cooling down to room temperature, each flask was inoculated with 20.0 mL of the spore and incubated at 25 °C for 40 days.

The fermented rice substrate was extracted repeatedly with EtOAc (3 × 50 L), and the organic solvent was completely evaporated under vacuum to afford the crude extract (579 g). The crude extract was then suspended into water (3 L) and partitioned with *n*-hexane (3 × 3 L) and EtOAc (3 × 3 L), successively. The EtOAc fraction (61 g) was subjected to CC over MCI-gel CHP20P, eluted with gradient mixture of MeOH and H_2_O (10:90–100:0, v/v), to give 11 fractions (Fr.1–Fr.11). Fr.10 (656 mg) was separated by silica gel CC, eluting with CHCl_3_–MeOH (100:1–20:1) to give five sub-fractions (Fr.10-1–Fr.10-5). Fr.10-2 (101 mg) was purified by semi-preparative HPLC (MeCN–H_2_O, 32: 68, v/v) to afford **3** (7.0 mg, t_R_ = 12.7 min) and **4** (23 mg, t_R_ = 21.6 min). Fr.10-3 (68 mg) was subjected to semi-preparative HPLC (MeCN–H_2_O, 18: 82, v/v) to afford **2** (2.1 mg, t_R_ = 19.9 min). Fr.10-4 (96 mg) was applied to semi-preparative HPLC (MeCN–H_2_O, 28: 72, v/v) to afford **1** (3.4 mg, t_R_ = 15.4 min).

Rigidiusculamide E (**1**): colorless oil; $[\alpha ]_{\text{D}}^{25}$ −61.8 (*c* 0.03, MeOH); UV (MeOH) λ_max_ nm (log ε): 471 (2.15), 362 (2.28), 286 (3.42), 228 (3.90), 203 (4.45); IR (KBr) ν_max_ cm^−1^: 3419, 2973, 2930, 1669, 1492, 1380, 1247, 1180; ^1^H and ^13^C NMR (CDCl_3_): see Table 1; Positive ESIMS: *m/z* 342 [M+Na]^+^.

[-(*α*-Oxyisohexanoyl-*N*-methyl-leucyl)_2_-] (**2**): colorless crystal; $[\alpha ]_{\text{D}}^{25}$ 8.8 (*c* 0.02, MeOH); UV (MeOH)λ_max_ nm (log ε): 292 (3.20), 205 (4.51); IR (KBr) ν_max_ cm^−1^: 2963, 2932, 2878, 1758, 1657, 1456, 1370, 1177, 1144; ^1^H and ^13^C NMR (CDCl_3_): see Table 1; Positive ESIMS: *m/z* 505 [M+Na]^+^.

### The Nitric Oxide Production in RAW264.7 Macrophages

Murine macrophage cell line RAW264.7 was obtained from Cell Bank of Chinese Academy of Sciences (Beijing, People’s Republic of China). RAW264.7 cells were seeded in 96-well cell culture plates (1.5 × 10^5^ cells/well) and treated with serial dilutions of the compounds with a maximum concentration of 25 μM in triplicate, followed by stimulation with 1 μg/mL LPS (Sigma, St. Louis, MO, USA) for 18 h. nitric oxide production in the supernatant was assessed by Griess reagents (Reagent A & Reagent B, respectively, Sigma) [22]. The absorbance at 570 nm was measured with a microplate reader (Thermo, Waltham, MA, USA). *N*^G^-Methyl-l-arginine acetate salt (L-NMMA, Sigma), a well-known nitric oxide synthase (NOS) inhibitor, was used as a positive control (half maximal inhibitory concentration IC_50_ = 39.41 ± 2.43 μM) [23]. All the compounds were prepared as stock solutions in DMSO. The viability of RAW264.7 cells was evaluated by the MTS assay simultaneously to exclude the interference of the cytotoxicity of the test compounds.

## Conclusions

Two new alkaloids, rigidiusculamide E (**1**) and [-(*α*-oxyisohexanoyl-*N*-methyl-leucyl)_2_-] (**2**), together with two known ones, (-)-oxysporidinone (**3**) and (-)-4,6′-anhydrooxysporidinone (**4**), were identified from *F. tricinctum* SYPF 7082, an endo-phytic fungus isolated from the root of *Panax notoginseng*. All of them were obtained from *F. tricinctum* for the first time. The new compound **2** showed inhibition of NO production in Murine macrophage cell line with the IC_50_ value of 18.10 ± 0.16 μM.

## Electronic supplementary material

Below is the link to the electronic supplementary material.

**Supplementary data:** 1D and 2D NMR, ESIMS, HRESIMS, IR, CD and UV spectra of compounds **1**–**2** are available as Supporting Information (SI). Supplementary material 1 (DOCX 5230 kb)
