# Supplementary material for: Two New Alkaloids from Fusarium tricinctum SYPF 7082, an Endophyte from the Root of Panax notoginseng
Source: Nat Prod Bioprospect. 2018 Jun 18;8(5):391–6. doi: 10.1007/s13659-018-0171-0 (PMC6109442; doi:10.1007/s13659-018-0171-0)

**Supplementary Material**

**Two new alkaloids from *Fusarium* *tricinctum* SYPF 7082, an endophyte from the root of *Panax notoginseng***

Wen-Jie Sun ^a,b^, Hong-Tao Zhu ^a^, Tian-Yuan Zhang ^c^, Meng-Yue Zhang ^c^, Dong Wang ^a^, Chong-Ren Yang ^a^, Yi-Xuan Zhang^*c^, and Ying-Jun Zhang^*a,d^

*^a^* *State Key Laboratory of Phytochemistry and Plant Resources in West China, Kunming Institute of Botany, Chinese Academy of Sciences, Kunming 650201, People’s Republic of China.*.

*^b^* *University of Chinese Academy of Sciences, Beijing 100049, People’s Republic of China.*

*^c^ Shenyang Pharmaceutical University, Shenyang 110016, People’s Republic of China*

^d^ *Yunnan Key Laboratory of Natural Medicinal Chemistry, Kunming Institute of Botany, Chinese Academy of Sciences, Kunming, People's Republic of China.*

**Corresponding authors**

*E-mail: [zhangyj@mail.kib.ac.cn](mailto:zhangyj@mail.kib.ac.cn) (Y.-J. Zhang)

*E-mail: [zhangyxzsh@163.com](mailto:zhangyxzsh@163.com) (Y.-X. Zhang).

Contents

**Table S1**. ^1^H (600 MHz) and ^13^C (150 MHz) NMR data of **1** (in CDCl_3_) and rigidiusculamide D (acetone-d_6_)…………………………………………………..……………………………………..3

**Table S2**. ^1^H (600 MHz) and ^13^C (150 MHz) NMR data of **3**-**4** …………………………………..4

**Figure S1**. ^1^H NMR (600 MHz, in CDCl_3_) spectrum of **1**………..………………………………..5

**Figure S2**. ^13^C NMR (150 MHz, in CDCl_3_) spectrum of **1**…………………………………..…….5

**Figure S3**. HMQC spectrum of **1**…………………………………………………………………..6

**Figure S4**. HMBC spectrum of **1**…………………………………………………………………..6

**Figure S5**. ^1^H-^1^H COSY spectrum of **1**………………………………………………………….…7

**Figure S6**. ROESY spectrum of **1**……………………………………….…………………………7

**Figure S7**. HRESIMS of **1**……………………..…………………………………..……………….8

**Figure S8**. UV spectrum of **1**………………………………………………………………….……9

**Figure S10**. ^1^H NMR (600 MHz, in CDCl_3_) spectrum of **2**………………………………..……..10

**Figure S11**. ^13^C NMR (150 MHz, in CDCl_3_) spectrum of **2**……………………………….……..10

**Figure S12**. HMQC spectrum of **2**……………………………………………………….…….…11

**Figure S13**. HMBC spectrum of **2**………………………………………………………………...11

**Figure S14**. ^1^H-^1^H COSY spectrum of **2**……………………….…………………………...…….12

**Figure S15**. ROESY spectrum of **2**………………………………………………………….……12

**Figure S16**. HRESIMS of **2**…….…………………………………………………..……………..13

**Figure S17**. UV spectrum of **2**……………………………………………………..…….………..14

**Figure S18**. IR spectrum of **2**..……………………………………………...…………………….14

# Table S1. ^1^H (600 MHz) and ^13^C (150 MHz) NMR data of 1 (in CDCl_3_) and rigidiusculamide D (acetone-*d*_6_) (*J* in Hz)

|  | **1** | | rigidiusculamide D | |
| --- | --- | --- | --- | --- |
| No. | δ_C_ | δ_H_ | δ_C_^a^ | δ_H_^b^ |
| 2 | 176.2, s |  | 173.2, s |  |
| 3 | 42.7, d | 2.35, m | 75.1, s |  |
| 4 | 69.1, d | 3.94, dd (9.0, 4.8) | 72.9, d | 3.69, d (5) |
| 5 | 64.5, d | 3.52, m | 63.1, d | 3.52, t (5) |
| 6 | 32.5, t | 2.95, dd (13.2, 4.2)  2.84, dd (13.2, 4.2) | 33.2, t | 3.00, dd (14, 5)  2.98, dd (14, 5) |
| 7 | 129.2, s |  | 130.5, s |  |
| 8 | 126.0, d | 7.06, s | 129.6, d | 7.24, s |
| 9 | 128.0, s |  | 128.5, s |  |
| 10 | 158.6, s |  | 159.6, s |  |
| 11 | 109.2, d | 6.66, d (8.4) | 109.2, d | 6.64, d (8.0) |
| 12 | 128.9, d | 6.98, d (8.4) | 126.8, d | 7.12, d (8.0) |
| 13 | 27.9, q | 2.80, s | 27.9, q | 2.76 s |
| 14 | 8.6, q | 1.13, d (7.2) | 21.6, q | 1.21, s |
| 15 | 30.9, t | 3.10, dd (15.7, 9.0)  3.24, dd (15.7, 9.0) | 31.1, t | 3.27,dd (16.0, 9.0)  3.24,dd (16,9.0) |
| 16 | 90.0, d | 4.53, t (9.6) | 90.2, d | 4.61, t (9.0) |
| 17 | 72.0, s |  | 71.3, s |  |
| 18 | 26.6, q | 1.28, s | 25.4, q | 1.27, s |
| 19 | 24.4, q | 1.15, s | 26.0, q | 1.24, s |

^a^ recorded in 150 MHz; ^b^recorded in 500 MHz.

# Table S2. ^1^H (600 MHz) and ^13^C NMR (150 MHz) data of 3-4 (in CDCl_3_, *J* in Hz)

|  | **3** | | **4** | |
| --- | --- | --- | --- | --- |
| No. | δ_C_ | δ_H_ | δ_C_ | δ_H_ |
| 2 | 163.4, s |  | 165.3 , s |  |
| 3 | 111.3, s |  | 109.4, s |  |
| 4 | 164.3, s |  | 168.5, s |  |
| 5 | 119.3, s |  | 119.1, s |  |
| 6 | 138.5, d | 7.61, s | 135.9, d | 7.68, s |
| 7 | 79.2, d | 4.91, dd (10.0, 2.0) | 73.6, d | 4.75, m |
| 8 | 31.6, t | 1.58, 1.93, m | 30.3, t | 1.66, m |
| 9 | 33.1, t | 1.43, 1.93, m | 34.1, t | 1.88, 1.31, m |
| 10 | 33.8, d | 1.77, m | 33.0, d | 1.65, m |
| 11 | 93.8, d | 3.53, d (10.2) | 93.8, d | 3.30, m |
| 12 | 132.2, s |  | 134.0, s |  |
| 13 | 138.9, d | 5.25, dd (12.0, 1.8) | 137.4, d | 5.11, m |
| 14 | 31.0, d | 2.57, m | 31.0, d | 2.34, m |
| 15 | 46.1, t | 1.08, 1.26, m | 46.4, t | 1.21, 1.08, m |
| 16 | 33.4, d | 1.33, m | 33.8, d | 1.41, m |
| 17 | 29.9, t | 1.08, 1.43, m | 30.0, t | 1.41, 1.08, m |
| 18 | 11.6, q | 0.86, d (7.2) | 11.7, q | 0.84, d (7.8) |
| 19 | 18.2, q | 0.79, d (6.6) | 18.2, q | 0.73, d (6.6) |
| 20 | 12.7, q | 1.68, d (2.0) | 11.8, q | 1.56, m |
| 21 | 21.4, q | 0.94, d (6.6) | 21.4, q | 0.99, d (6.6) |
| 22 | 20.4, q | 0.86, d (7.2) | 20.2, q | 0.85, d (7.8) |
| 23 | 38.0, q | 3.49, s | 38.7, q | 3.50, s |
| 1' | 75.0, s |  | 77.2, s |  |
| 2' | 32.8, t | 1.74, 2.35, m | 34.4, t | 2.50, 2.17, m |
| 3' | 38.0, t | 1.68, 1.74, m | 35.9, t | 2.34, 2.24, m |
| 4' | 69.5, d | 3.66, m | 210.3, s |  |
| 5' | 33.4, t | 1.74, 2.01, m | 49.4, t | 3.03, dd (21.0, 4.2)  2.78, dd (21.0, 4.2) |
| 6' | 70.6, d | 4.29, dd (11.0,4.5) | 92.0, d | 4.93,d (4.0) |

# Figure S1. ^1^H NMR (600 MHz, in CDCl_3_) spectrum of 1

**
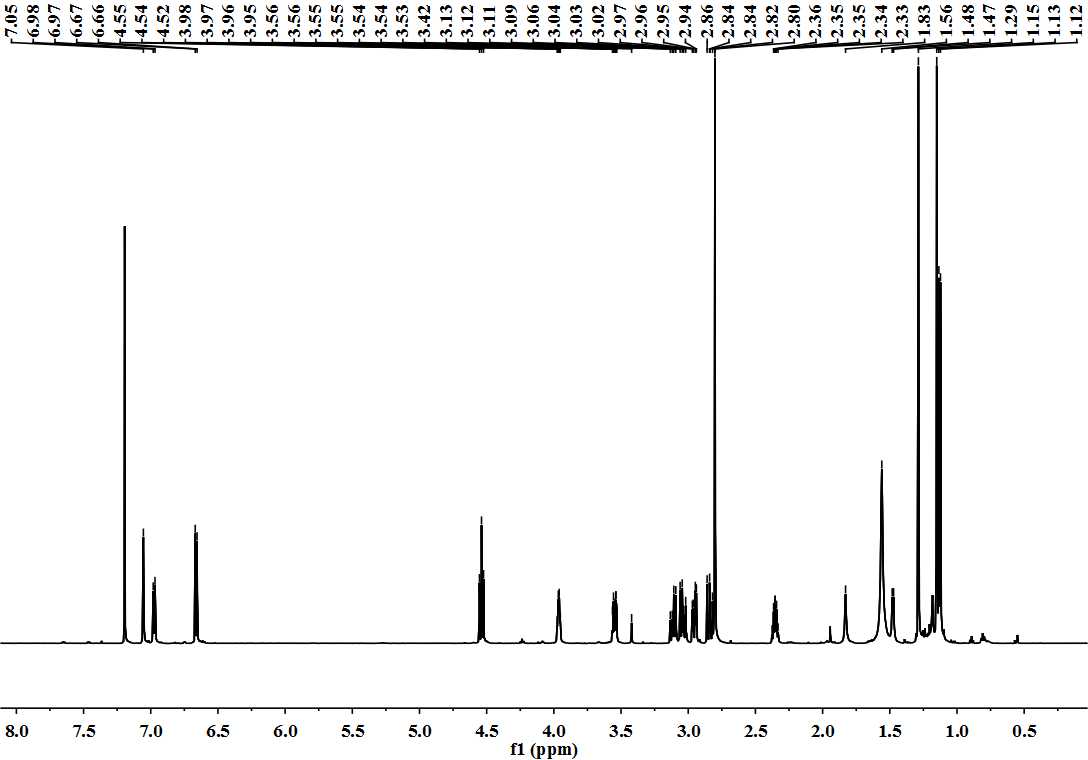
**

# Figure S2. ^13^C NMR (150 MHz, in CDCl_3_) spectrum of 1

**
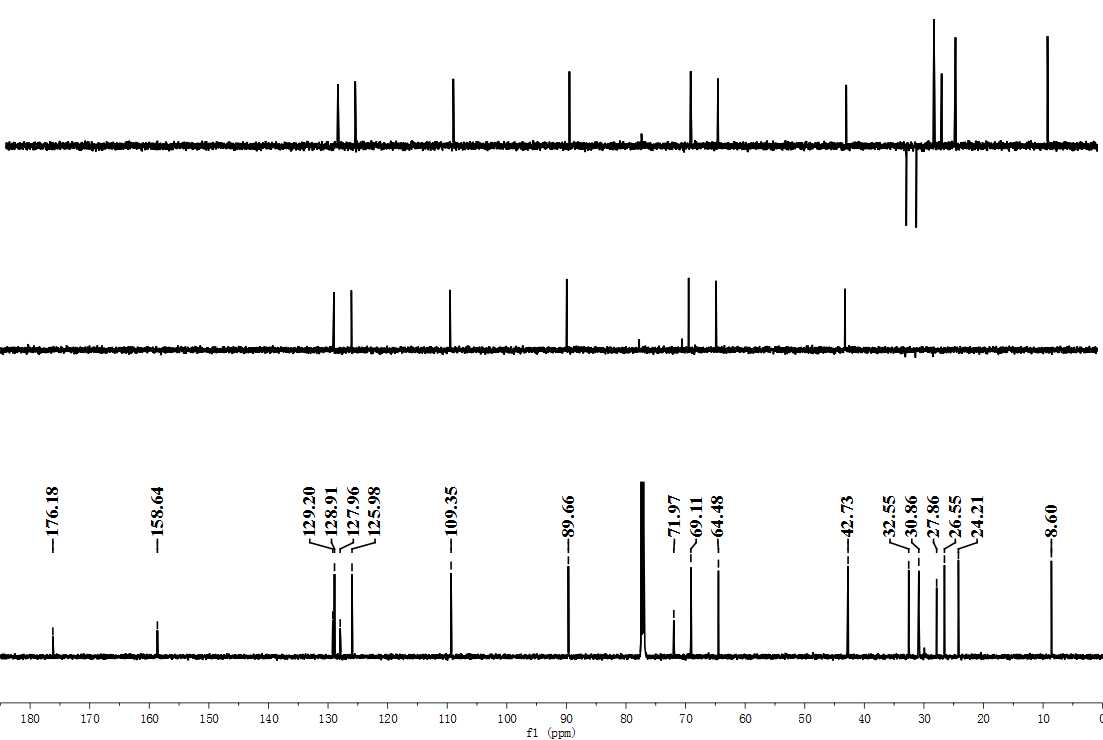
**

# Figure S3. HMQC spectrum of 1

#
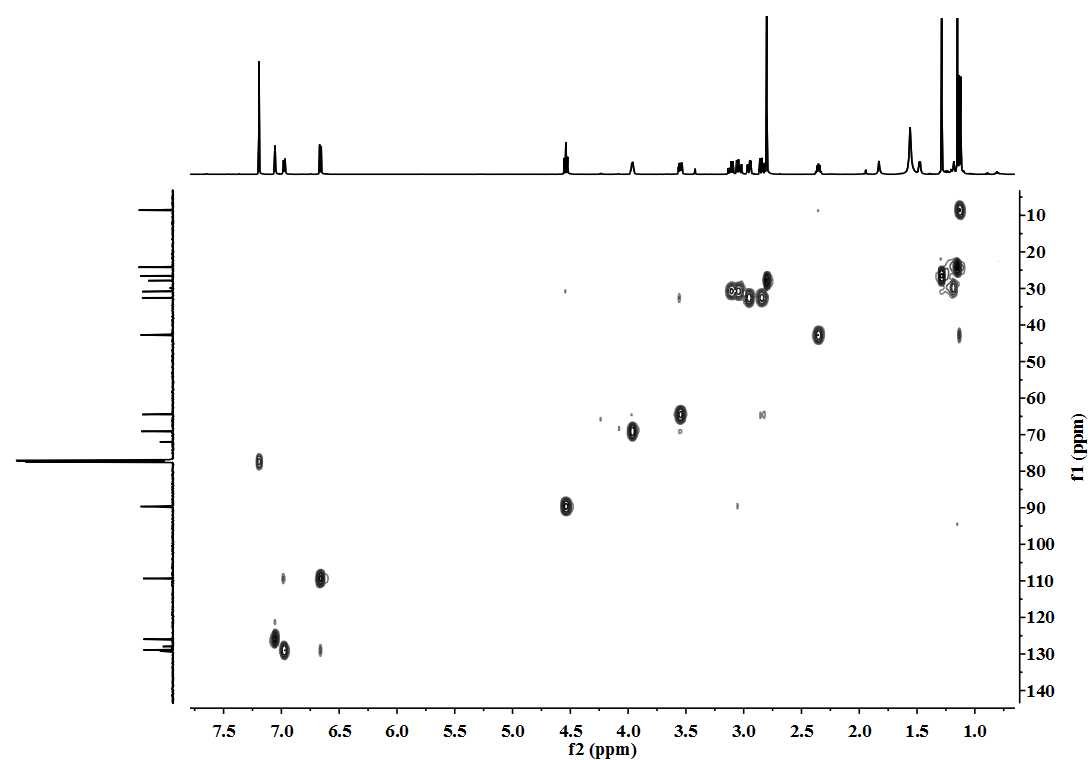


# Figure S4. HMBC spectrum of 1

**
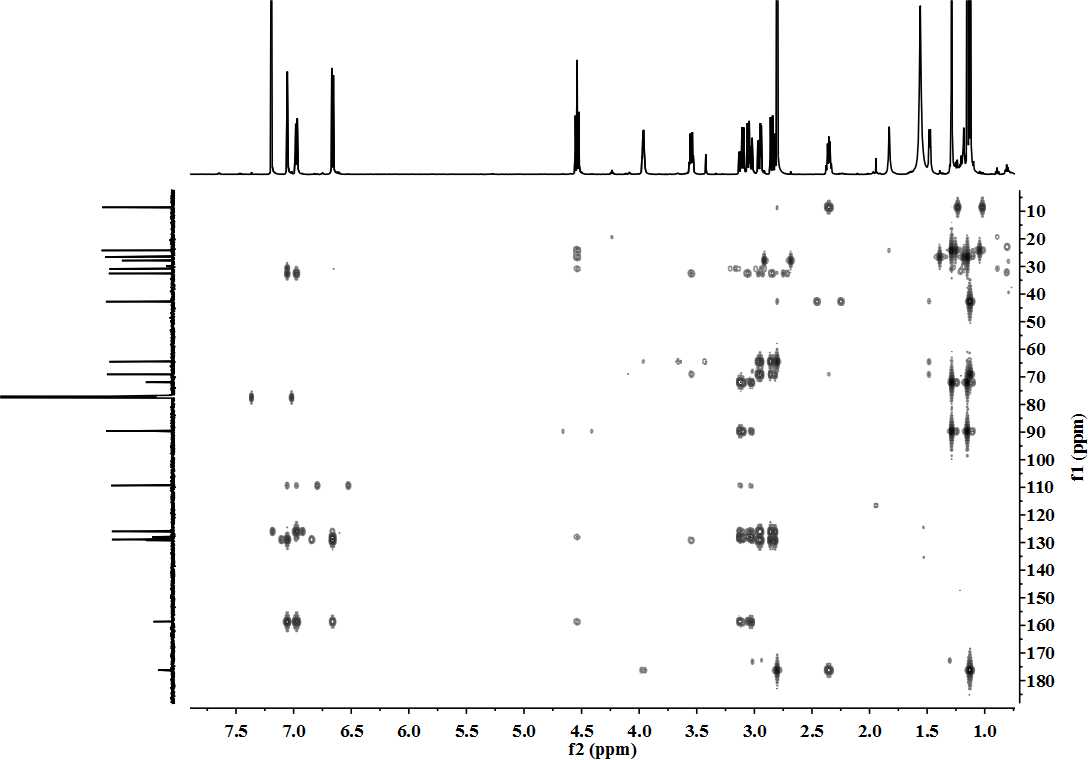
**

# Figure S5. ^1^H-^1^H COSY spectrum of 1

**
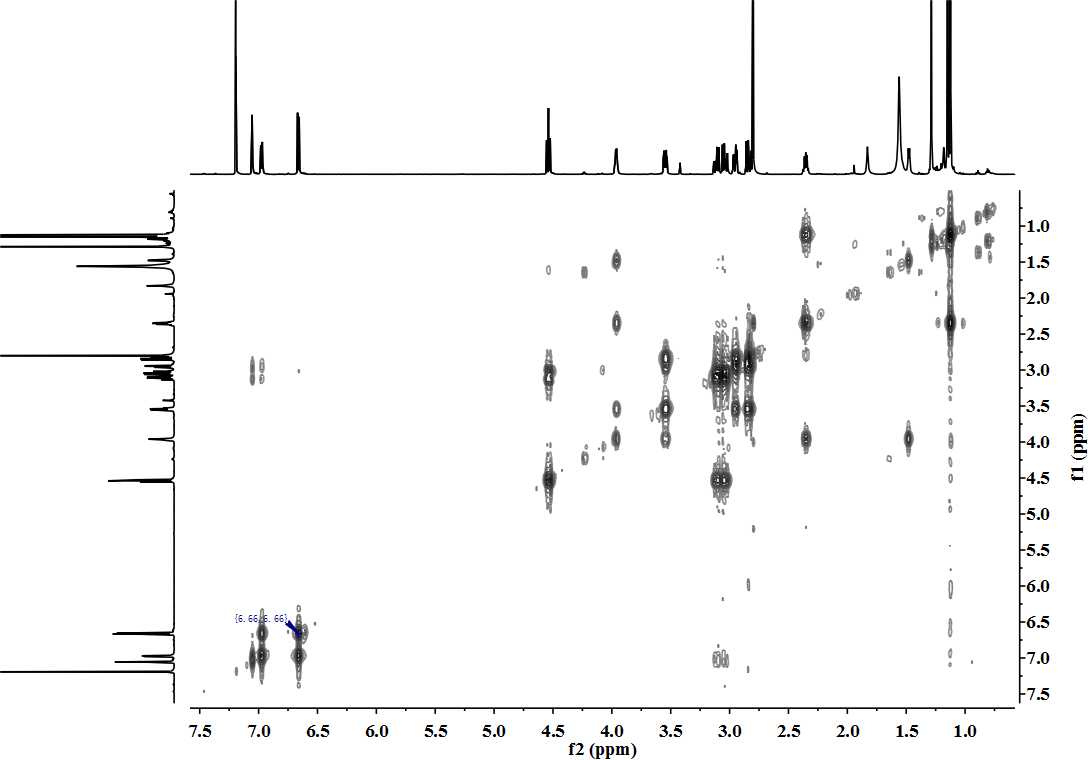
**

# Figure S6. ROESY spectrum of 1


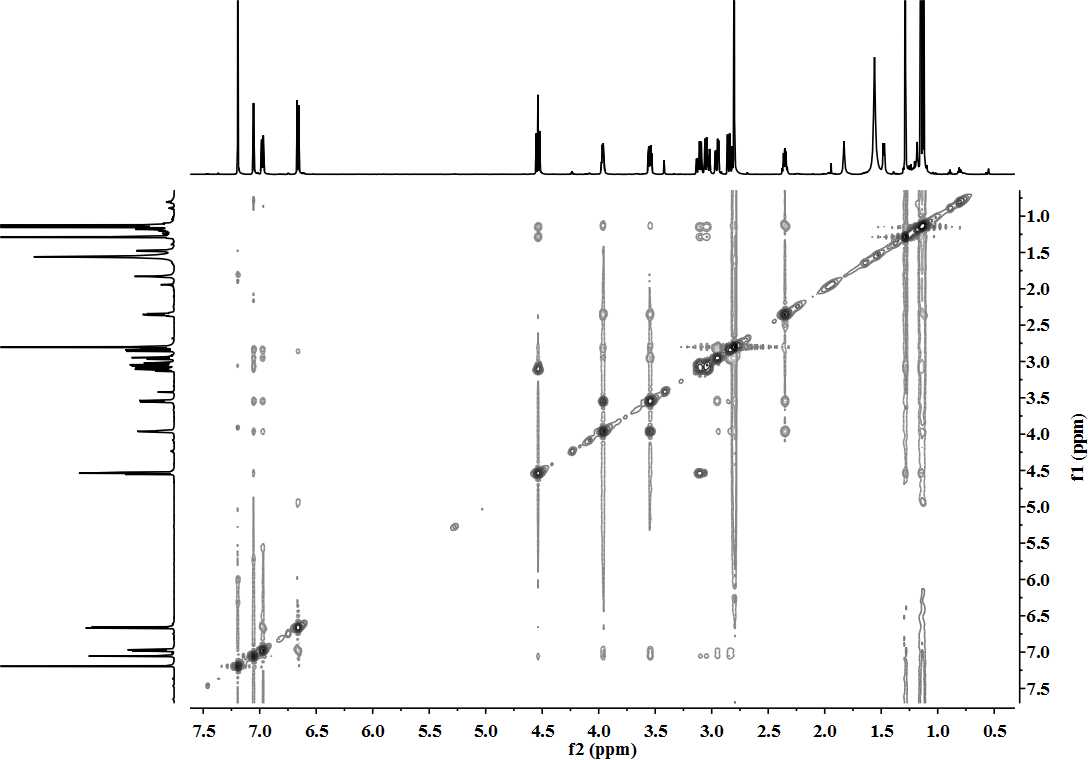


# Figure S7. HRESIMS of 1


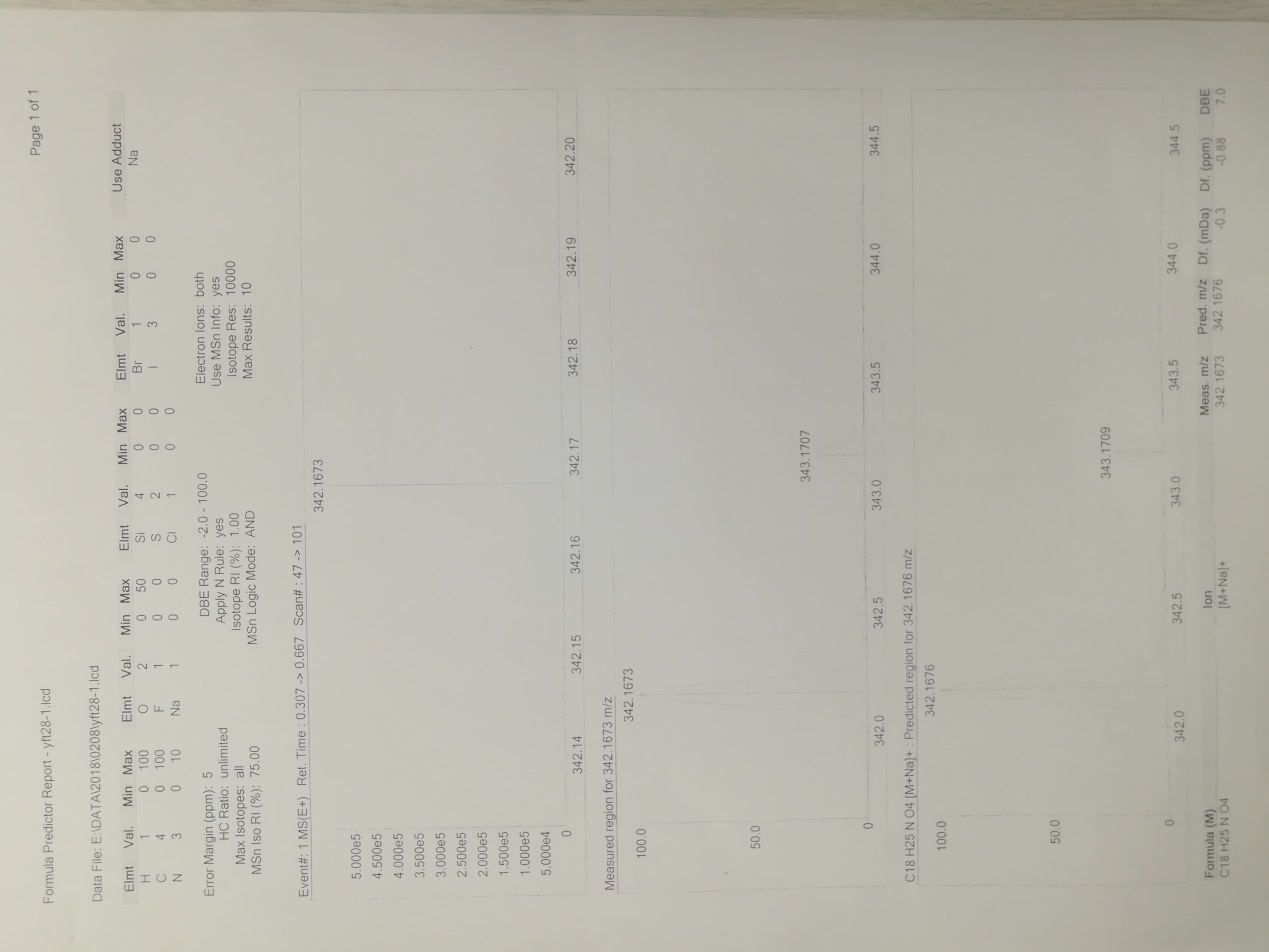


# Figure S8. UV spectrum of 1

**
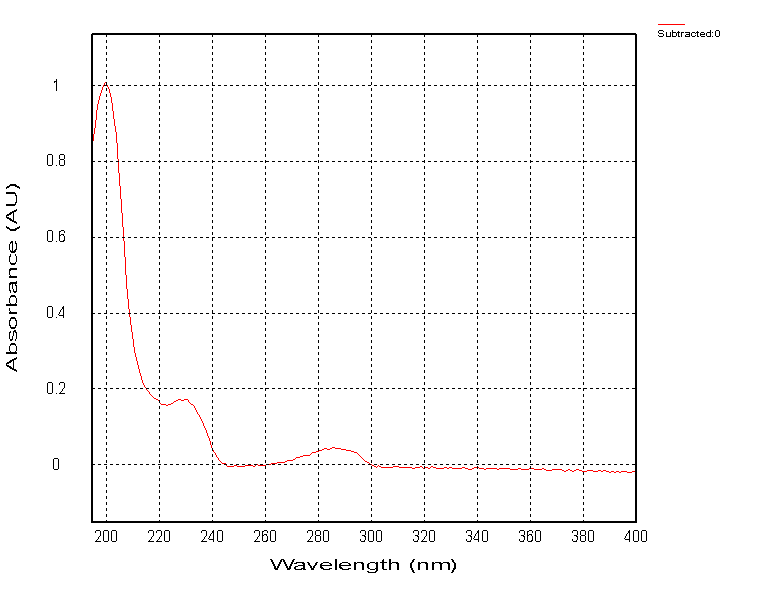
Figure S9**. IR spectrum of **1**


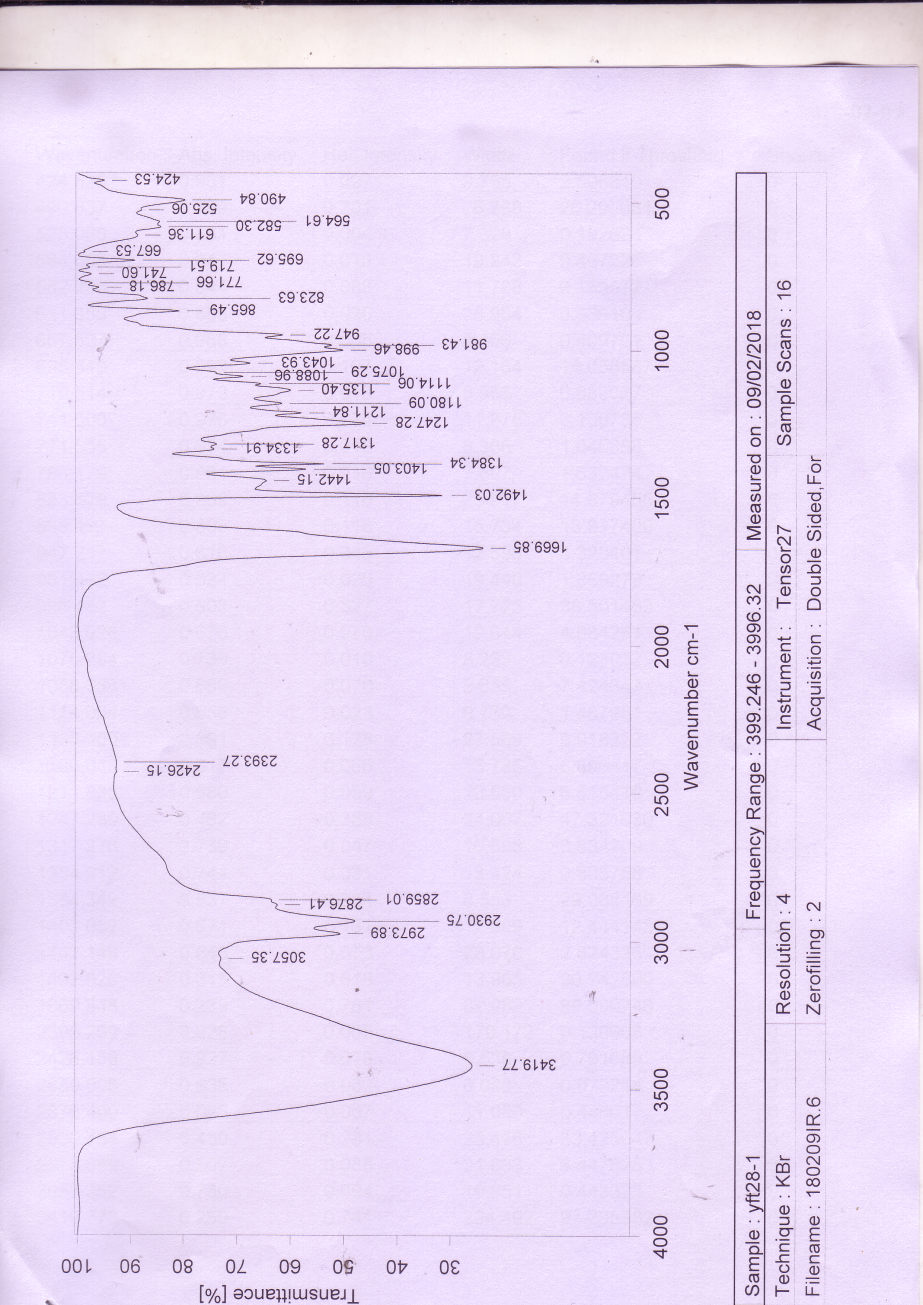


# Figure S10. ^1^H NMR (600 MHz, in CDCl_3_) spectrum of 2


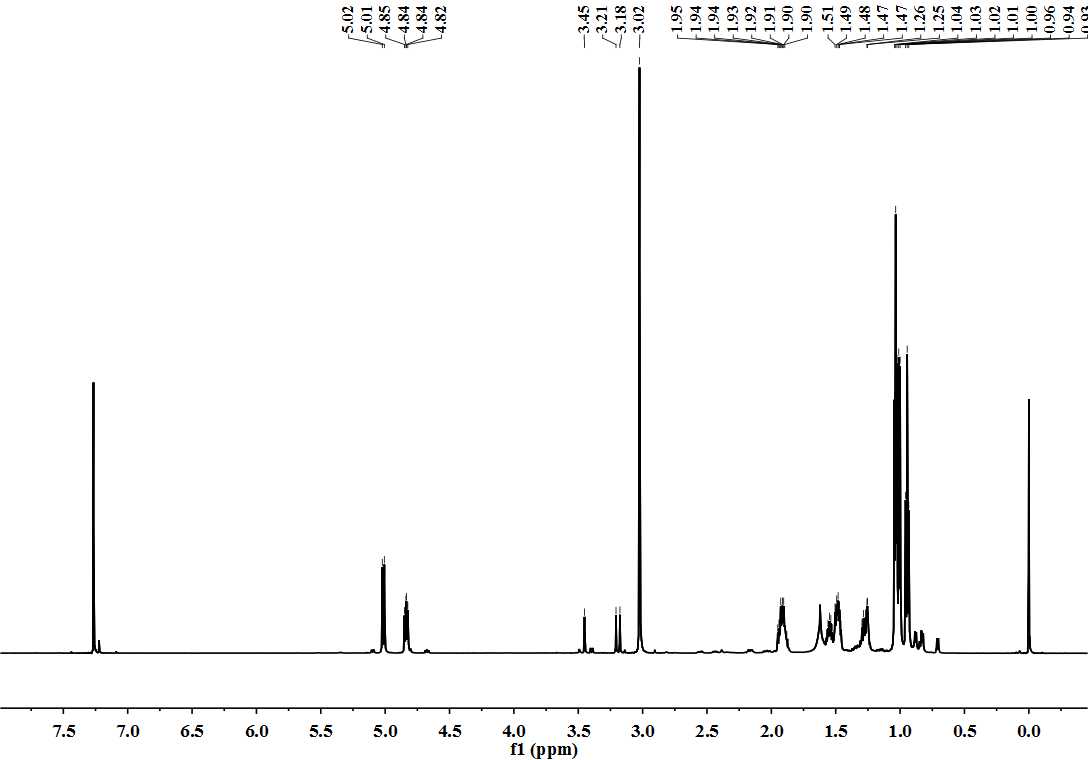


# Figure S11. ^13^C NMR (150 MHz, in CDCl_3_) spectrum of 2

**
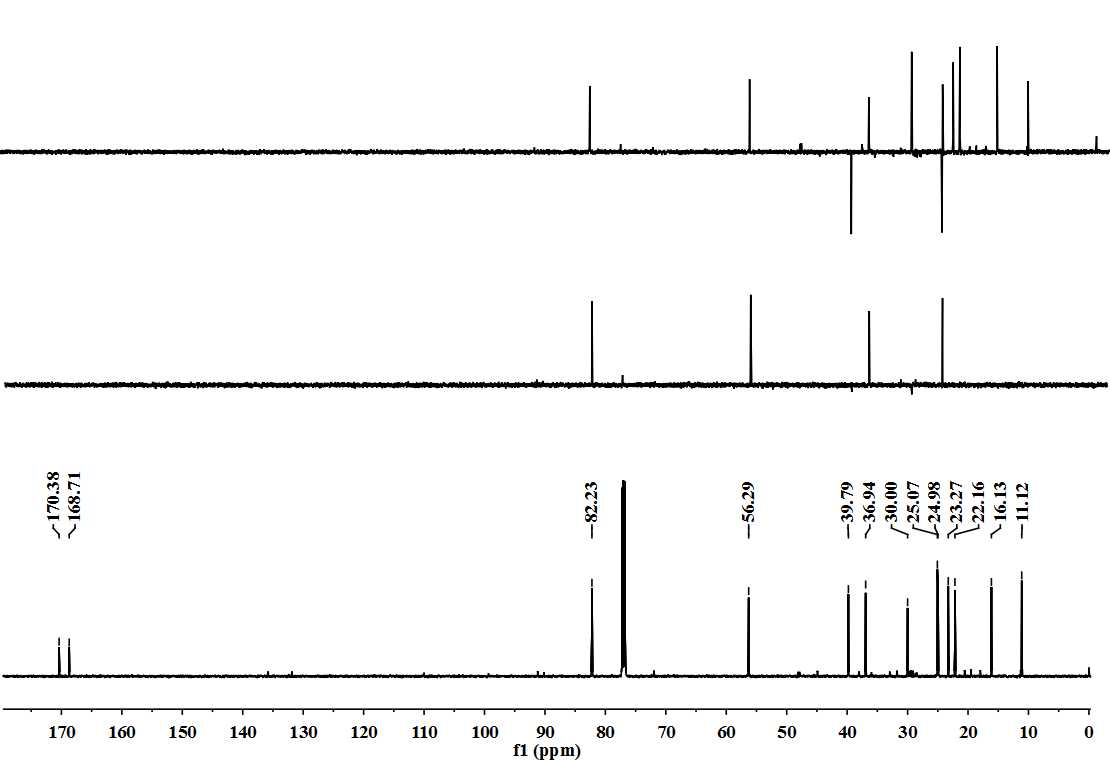
**

**Figure S12**. HMQC spectrum of 2


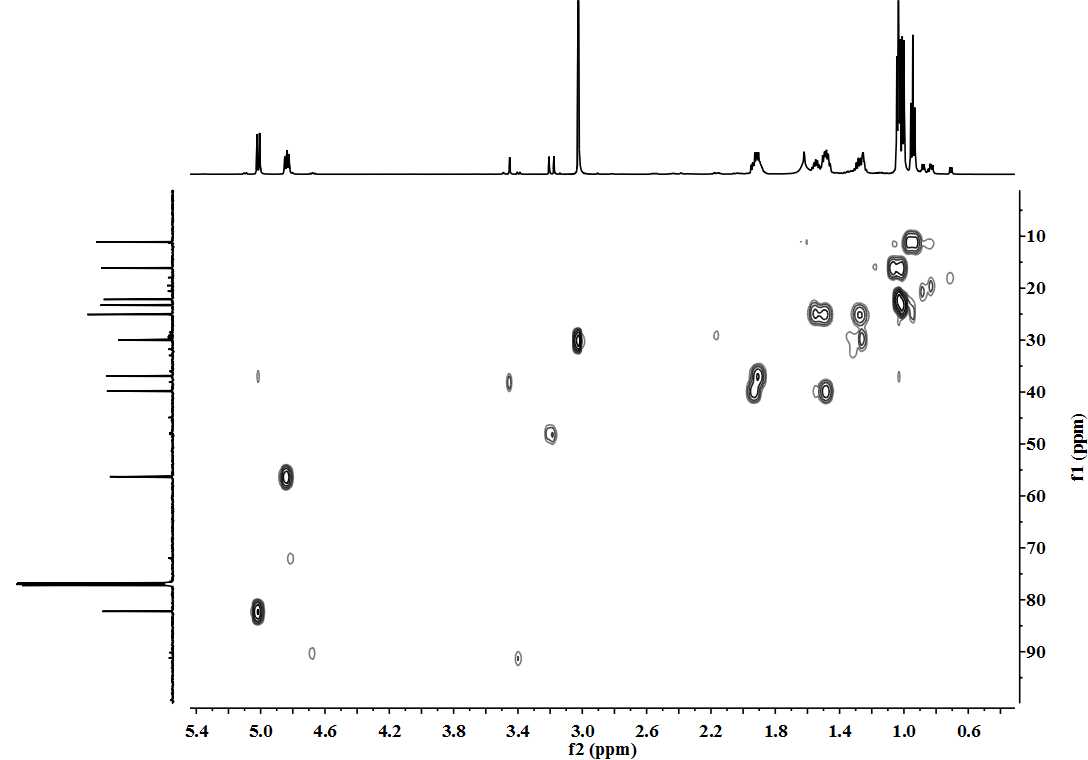


# Figure S13. HMBC spectrum of 2


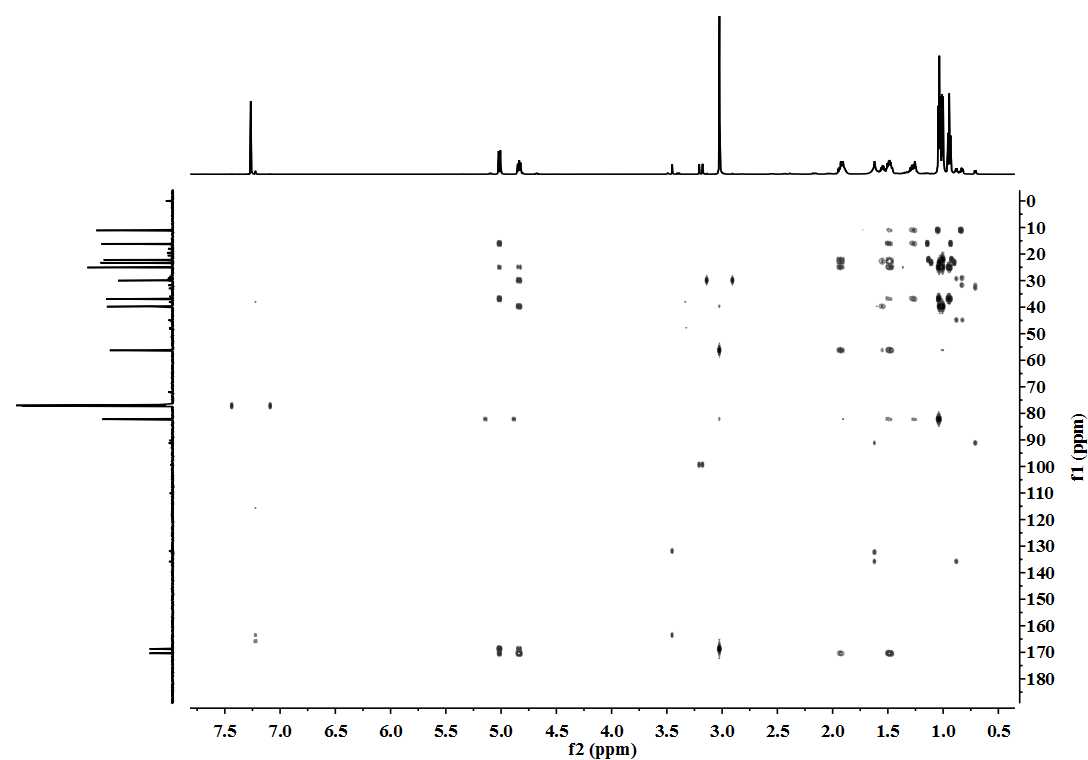


# Figure S14. ^1^H-^1^H COSY spectrum of 2

**
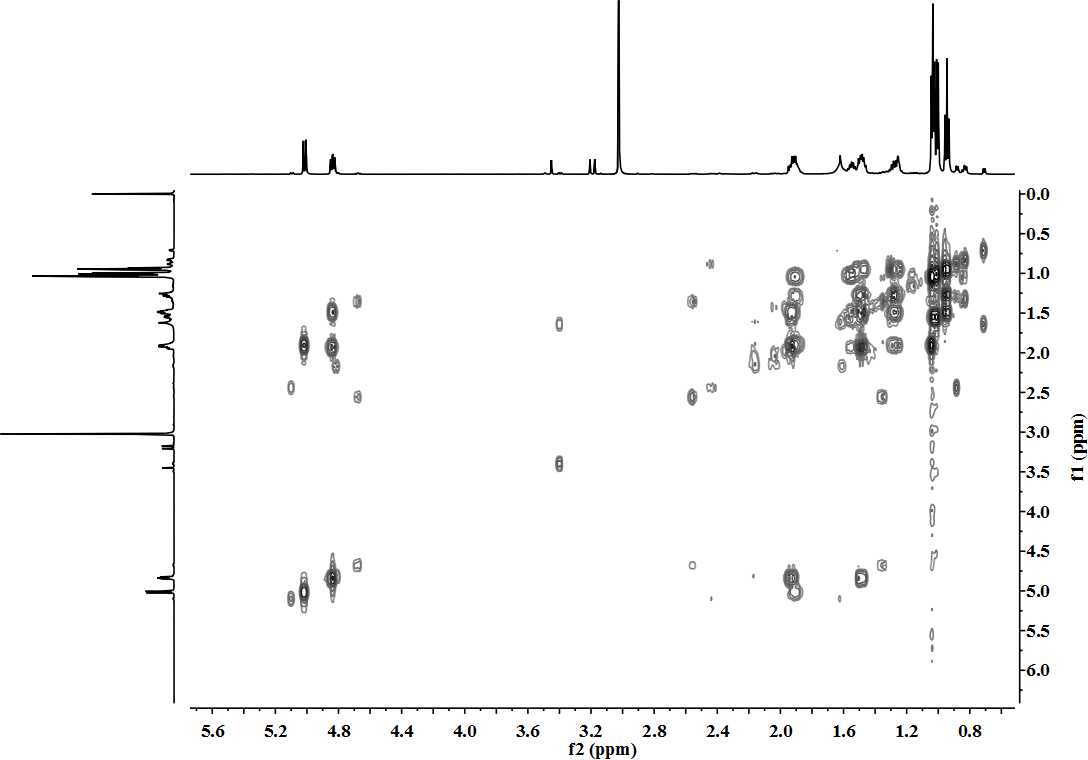
**

# Figure S15. ROESY spectrum of 2


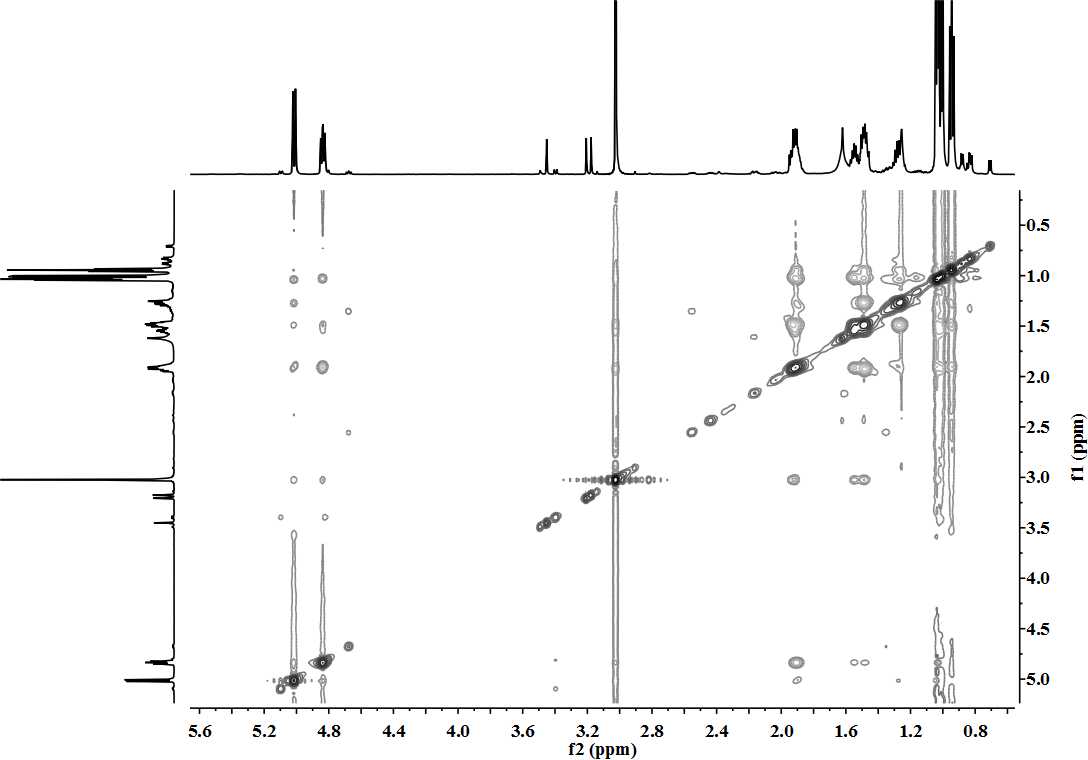


# Figure S16. HRESIMS of 2


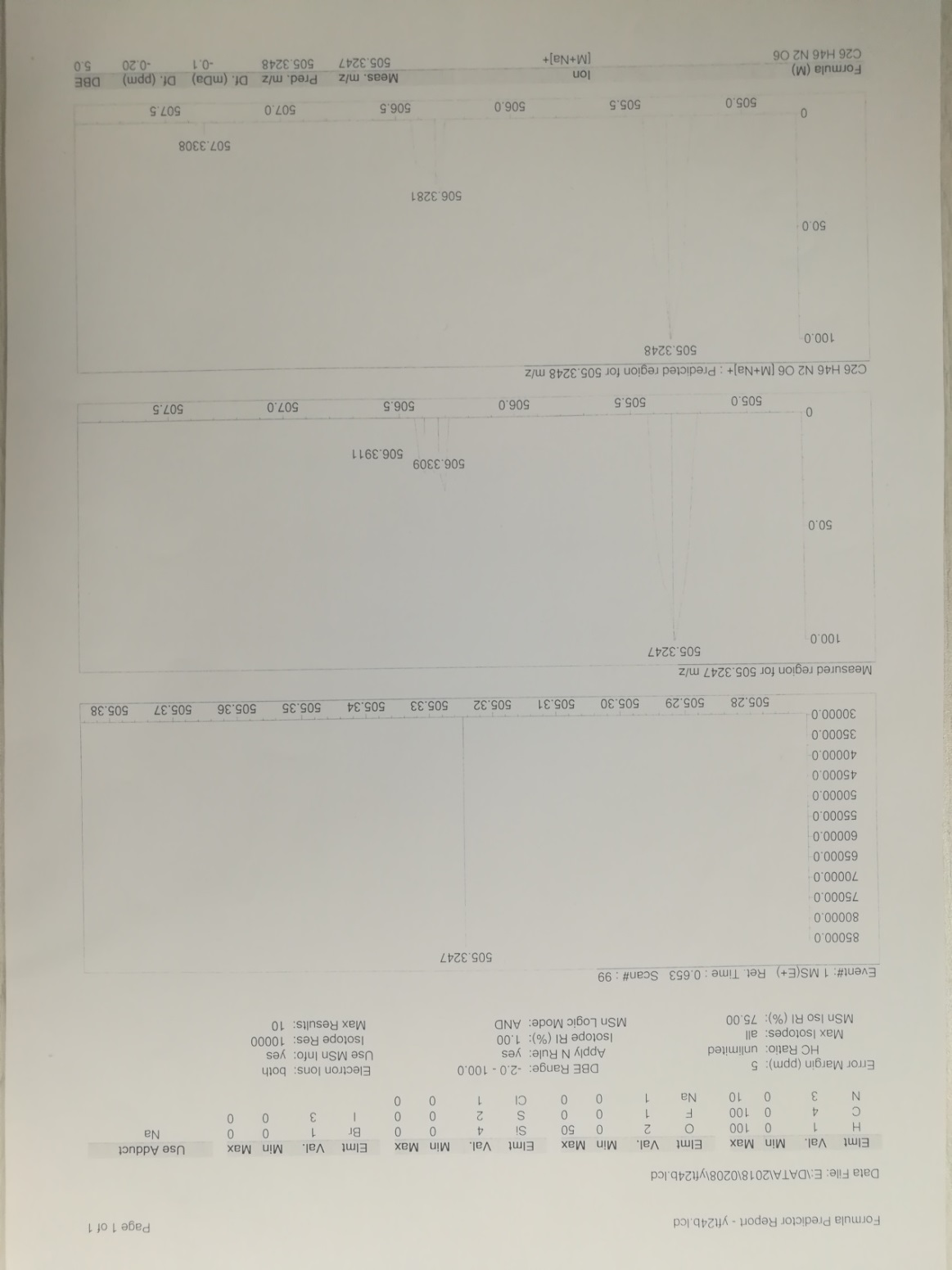


# Figure S17. UV spectrum of 2

**
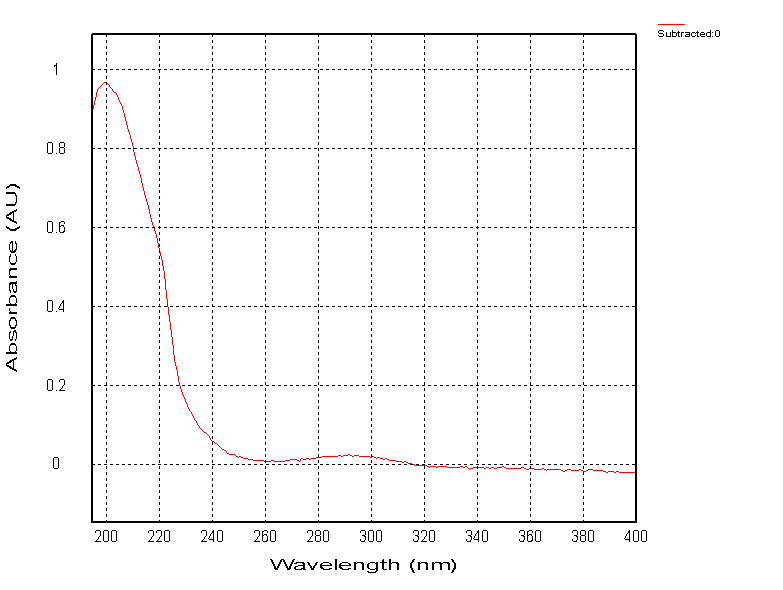
**

# Figure S18. IR spectrum of 2


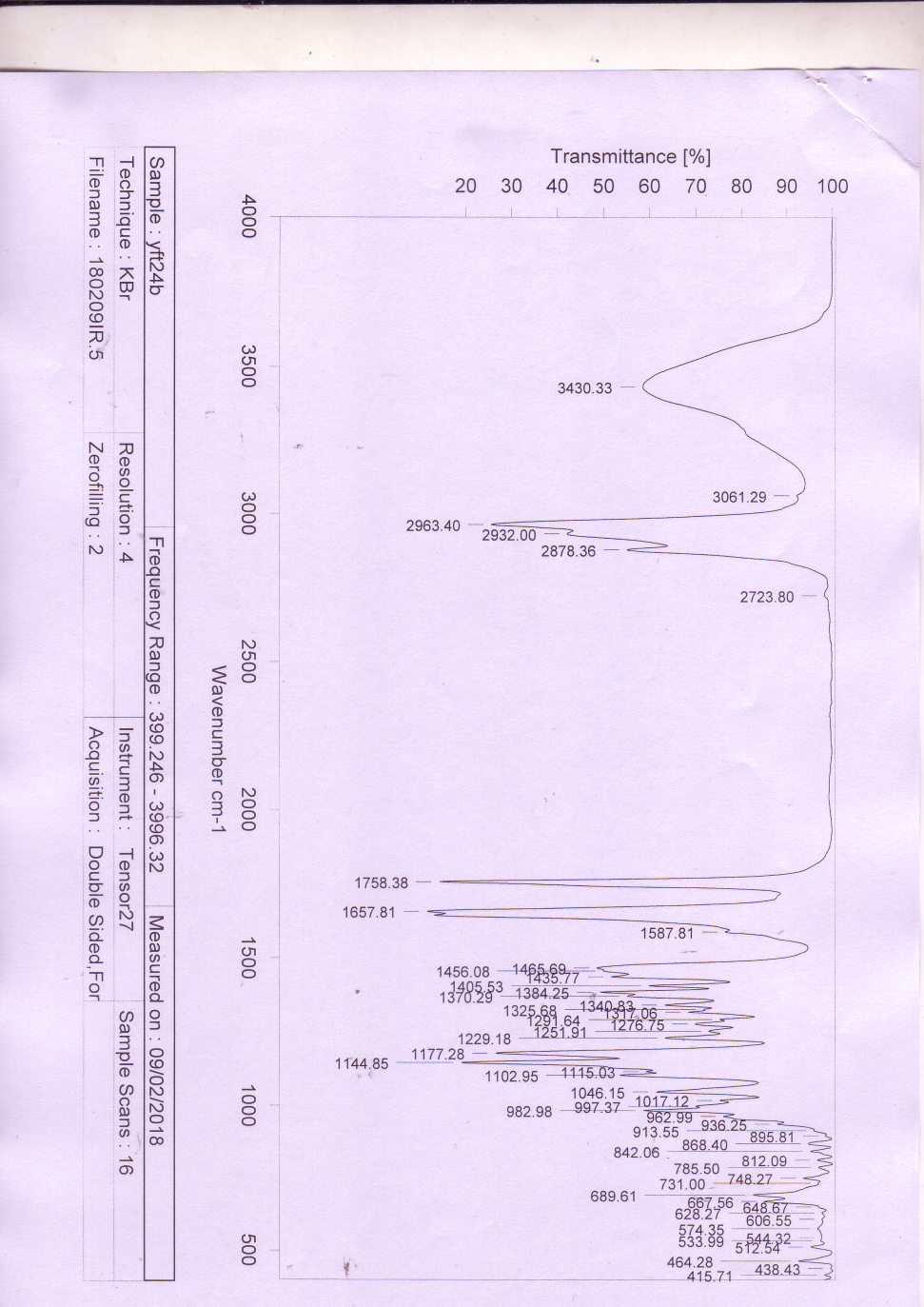

Supplement: Supplementary file 1 — Supplementary data: 1D and 2D NMR, ESIMS, HRESIMS, IR, CD and UV spectra of compounds 1–2 are available as Supporting Information (SI). Supplementary material 1 (DOCX 5230 kb) [file 13659_2018_171_MOESM1_ESM.docx]
